# The role of *Piper chaba* Hunt. and its pure compound, piperine, on TRPV1 activation and adjuvant effect

**DOI:** 10.1186/s12906-020-02917-4

**Published:** 2020-05-05

**Authors:** Sumalee Panthong, Yasuyuki Imai, Takeshi Matsuoka, Wakana Suzuki, Tatsuo Watanabe, Yuko Terada, Kohta Kurohane, Kota Sekiguchi, Erina Ogawa, Yukina Endo, Arunporn Itharat

**Affiliations:** 1 Department of Applied Thai Traditional Medicine, Faculty of Medicine, Thammasat University, Klongluang, Pathumthani, 12120 Thailand; 2 Centre of Excellence in Applied Thai Traditional Medicine Research (CEATMR), Thammasat University, Klongluang, Pathumthani, 12120 Thailand; 3 Laboratory of Microbiology and Immunology, School of Pharmaceutical Sciences, University of Shizuoka, Shizuoka-shi, Shizuoka, 422-8526 Japan; 4 Laboratory of Food Chemistry, School of Food and Nutritional Sciences|, University of Shizuoka, Shizuoka-shi, Shizuoka, 422-8526 Japan

**Keywords:** *Piper. chaba* Hunt., Piperine, Contact hypersensitivity, TRPV1 activation

## Abstract

**Background:**

*Piper chaba* Hunt. is used as an ingredient in Thai traditional preparation for arthritis. Its isolated compound is piperine which shows anti-inflammatory activity. Piperine produces a burning sensation because it activates TRPV1 receptor. The TRPV1 activation involved with the analgesic and adjuvant effect. *P. chaba* Hunt. has not been reported about TRPV1 activation and adjuvant effect. The aim of this study was to investigate the effect of *P. chaba* extract and piperine on TRPV1 receptor, which is considered as a target for analgesic and their adjuvant effects to support the development of an analgesic drug from herbal medicine.

**Methods:**

The effect of *P. chaba* extract and piperine on HEK cells expressing TRPV1 channel was examined by calcium imaging assay. Adjuvant effects of *P. chaba* extract and piperine were investigated by a fluorescein isothiocyanate (FITC)-induced contact hypersensitivity (CHS) model in mice.

**Results:**

*P. chaba* extract induced calcium influx with EC_50_ value of 0.67 μg/ml. Piperine induced calcium influx with EC_50_ value of 0.31 μg/ml or 1.08 μM. For mouse CHS model, we found that 1% piperine, 5% piperine, 1% *P. chaba* extract and 5% *P. chaba* extract significantly enhanced sensitization to FITC as revealed by ear swelling responses.

**Conclusion:**

*P. chaba* extract and piperine activated TRPV1 channel and enhanced contact sensitization to FITC.

## Background

*Piper chaba* Hunt. (Thai as Dee-Plee) is a plant in the Piperaceae family. *P. chaba* Hunt. is used as an ingredient in Thai traditional preparation for arthritis [1]. The ethanolic extract of *P. chaba* Hunt. fruits is anti-inflammatory, analgesic and antipyretic as reported in an animal study [2].

*P. chaba* Hunt. contains pungent alkaloids such as piperine [3]. Piperine has also shown anti-inflammatory, antinociceptive, antiarthritic, anti-tumor and anti-bacteria activities [4–8]. Piperine’s taste is sharp, peppery and leaves a burning sensation because it is reported to activate human transient receptor potential cation channel subfamily V member 1 (TRPV1) which is similar to the capsaicin effect [9]. Moreover, piperine can modulate another ion channel such as GABA_A_ receptor [10].

TRPV1 or capsaicin receptor is a cation channel which consists of six transmembrane domains. It is activated by capsaicin, noxious heat, low pH and voltage [11]. The TRPV1 is involved in sensory neural response such as pain and pungency [12]. The TRPV1 channel is activated by many herbal substances such as eugenol, gingerol, resiniferatoxin, camphor and piperine [13]. The TRPV1 activation produces Na^+^ and Ca^2+^ influx into cells. The Ca^2+^ influx is important for the response to the pain and the desensitization to painful sensations over time [14]. For example, capsaicin is used as an analgesic drug which can activate TRPV1 and prolonged activation leads to desensitization of TRPV1 [15, 16]. It has been suggested that TRPV1 activation and desensitization are related to be analgesic and counterirritant effects [17].

During studies of contact hypersensitivity to fluorescein isothiocianate (FITC) in mice, several phthalate esters were found to enhance skin sensitization to FITC hapten [18]. As a mechanism of the enhancement, involvement of transient receptor potential ankyrin 1 (TRPA1) channel has been revealed [19]. This is supported by the results that some natural products of TRPA1 agonist, including cinnamaldehyde, menthol and carvacrol, exhibited adjuvant effects [20]. Despite the involvement of TRPV1 channels in the adjuvant effect [19], natural products with TRPV1 agonistic activity have not been tested for the adjuvant effect on contact sensitization.

Furthermore, TRPV1 activation and adjuvant effect of *P. chaba* Hunt. have never been reported. The aim of this study was to investigate the effect of *P. chaba* extract and its main compound as piperine on TRPV1 receptor and contact hypersensitivity in mice. The findings may be used for development of an analgesic drug from this plant in the future.

## Methods

### Reagents and chemicals

Acetone, AMG9810, dibutylphthalate (DBP) and dimethyl sulfoxide (DMSO) were obtained from Wako Pure Chemical Industries, Ltd. (Osaka, Japan). Blasticidin, zeocin and tetracyclin were purchased from Life Technologies (Carlsbad, CA, USA). Dulbecco’s modified Eagle’s medium (DMEM) and HAM’s F12 were purchased from Nissui Pharmaceuticals (Tokyo, Japan). Ethanol was purchased from RCI Labscan Limited (Bangkok, Thailand). Fetal bovine serum (FBS) was obtained from Hyclone (South Logan, UT). Fluorescein-4-isothiocyanate (FITC), Fluo 4-AM and HEPES were obtained from Dojindo Laboratories (Kumamoto, Japan). Ionomycin was obtained from LKT Laboratories Inc. (St. Paul, MN, USA). L-Glutamine was purchased from Nacalai Tesque., INC. (Kyoto, Japan). Pentobarbital sodium was obtained from Kyoritsu Seiyaku Corporation (Tokyo, Japan). Piperine was purchased from TCI Co., LTD. (Tokyo, Japan). Probenecid and Poly-L-lysine were obtained from Sigma (St. Louis, MO, USA). EDTA was purchased from KANTO Chemical Co., INC. (Tokyo, Japan).

### Plant material and preparation of plant extract

The fruits of *Piper chaba* Hunt. were collected by a Thai traditional doctor from Amphor Kaosaming, Chantaburi Province, Thailand in June 2010. It was identified and confirmed as *P. chaba* Hunt. by comparison with authentic voucher specimen number SKP 146160301 which is kept in the herbarium of the Southern Center of Thai Medicinal Plants, Faculty of Pharmaceutical Sciences, Prince of Songkla University, Songkla, Thailand. The fruits of *P. chaba* Hunt. were cleaned, cut into small pieces and dried at 50 °C. It was then ground into powder and 1 kg of plant powder was macerated with 95% ethanol for 3 days at room temperature, and filtered (Whatman No.1 paper). The filtrate was evaporated to dryness by a lyophilizer. The yield of ethanolic extract was 17.9%.

### Determination of piperine in the ethanolic extract of P. chaba Hunt. by HPLC

**Fig. 1:**
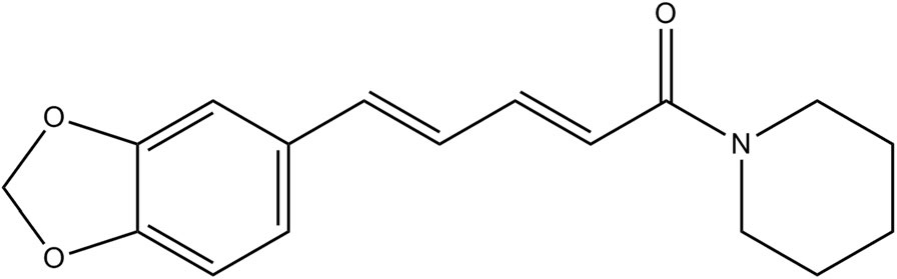
Chemical structure of piperine

Determination of the amount of piperine in *P. chaba* extract (PC extract) was carried out using High Performance Liquid Chromatography (HPLC) system (Constametric® 4100 Bio) with ultraviolet visible (UV-vis) detector (Spectromonitor® 4100) and automatic injector (Spectra System AS3500). Data was analyzed by TSP PC1000 software. A reversed-phase column, Phenomenax Luna 5 μ C18 [2] 100A analytical column (250 × 4.60 mm 5 μm) with guard column of the same material was used. The mobile phase consisted of water-acetonitrile with gradient elution as follows: 0 min, 60:40; 30 min, 50:50; 50 min, 5:95; 60 min, 0:100. The flow rate was 1 ml/min with UV absorbance detection at 256 nm. The piperine content in PC extract, which was calculated by standard curve of piperine (*R*^2^ = 0.998), was 194.10 mg/g of PC extract or 19.41%. The chemical structure of piperine is shown in Fig. 1.

### Cell and cultures

HEK293 cells stably expressing human TRPV1 were established with T-Rex system as described previously [21]. The cells were provided by Professor Tatsuo Watanabe (Laboratory of Food Chemistry, School of Food and Nutritional Sciences, University of Shizuoka). TRPV1-expressing HEK293 cells were cultured in Ham’s F-12 medium/DMEM (1:1) with 10% FBS, 10 mM HEPES, 60 μg/ml kanamycin sulfate, 200 μg/ml zeocin and 5 μg/ml blasticidin. Cells were sub-cultured every 3 days.

### Animals

Female BALB/c mice (7 weeks old) were obtained from Japan SLC Inc. (Shizuoka, Japan) and held for 1 week before use. The animals were maintained in standard environment conditions (23 ± 1 °C, 50–60% humidity) under a 12/12 h dark/light cycle. They had free access to standard food (Oriental Yeast Co., Tokyo, Japan) and water.

### Ethics and consent to participate

Experimental procedures were carried out in accordance with the Law concerning the Protection and Control of Animals, and the guidelines of Japan Ministry of Education, Culture, Sports, Science and Technology (MEXT) and those of the University of Shizuoka. The experimental protocols were approved by the Institutional Animal Care and Use Committee of the University of Shizuoka (approval number: 146135).

### Calcium imaging assay

TRPV1-expressing HEK293 cells or T-REx HEK293 cells were used for calcium imaging assay. T-Rex HEK293 cells or HEK293 cells transfected with tetracyclin repressor gene were used as control. T-Rex HEK293 cells were transfected by TRPV1 tranfection with vector pcDNA™4/TO/lacZ that were called TRPV1-expressing HEK293 cells and used as target cell. TRPV1-expressing HEK293 cells or T-REx HEK293 cell (4 × 10^4^ cells/well) were seeded into 96-well black plates coated with poly-L-lysine and incubated with 1 μg/ml of tetracyclin for 24 h at 37 °C in a humidified atmosphere containing 5% CO_2_. After incubation, cells were replaced with 3 μM Fluo-4-AM in loading buffer with 250 mM probenecid for 60–90 min at 37 °C. Cells were washed with 100 μl loading buffer and then 180 μl loading buffer was added into each well. The intracellular Ca^2+^ concentration was evaluated by Flex Station II (Molecular Devices, Sunnyvale, CA). After start of measurement, sample was added into each well at 30 s and 5 μM ionomycin was added at 150 s to measure the maximum level of calcium. Each sample was dissolved in DMSO and added to the loading buffer. The final concentration of DMSO did not exceed 0.1%. The cells were treated with various concentrations of capsaicin (1 nM-1 mM), piperine (30 nM-30 μM) or PC extract (0.027-11 μg/ml). Inhibitory activities of TRPV1 antagonist were performed by adding AMG9810 at various concentrations (30 nM, 300 nM and 3 μM) to piperine and PC extract. The results of each sample were expressed as the percentage response to 5 μM ionomycin. Dose-response curves and EC_50_ values were plotted and calculated with Prism 5.0 software (Graph Pad Software, San Diego, CA, USA).

### Sensitization and detection for contact hypersensitivity reaction

Experiments were performed as described previously with some modifications [18]. Fifty female BALB/c mice were divided into 9 groups: group 1: untreated group (5 mice), group 2: Acetone group for PC (6 mice), group 3: 1% PC extract in acetone (6 mice), group 4: 5% PC extract in acetone (6 mice), group 5: 2% DBP in acetone for PC (6 mice), group 6: Acetone group for piperine (5 mice), group 7: 1% piperine in acetone (5 mice), group 8: 5% piperine in acetone (5 mice), group 9: 2% DBP in acetone for piperine (6 mice). Mouse forelimbs were shaved 2 days before sensitization. The mice were anesthetized by an intraperitoneal injection of pentobarbital sodium (50 mg/kg). Mice were sensitized on days 0 and 7 with 160 μl of 0.5%FITC dissolved in acetone containing a test sample. The sample solutions were as follows: Acetone alone, 1% piperine in acetone, 5% piperine in acetone, 1% PC extract in acetone, 5% PC extract in acetone, 2% DBP in acetone. On day 14, the baseline ear thickness at 0 h was measured with a dial thickness gauge (Mitutoyo, Kanagawa, Japan). After that, mice were challenged by painting 20 μl of 0.5% FITC in 10%DBP-acetone on the right auricle, while 20 μl of 10% DBP-acetone was applied on the left auricle as a control. Mouse ear thickness was measured at 24, 48 and 72 h after challenge. In some experiments, mice without FITC-sensitization were challenged with FITC to serve as a negative control.

Ear swelling at X hrs is calculated as formula: [(ear thickness of the right ear at X hrs) – (ear thickness of the left ear at X hrs)] – [(ear thickness of the right ear at 0 h) – (ear thickness of the left ear at 0 h)] . Statistical analysis was performed using ANOVA and Tukey multiple comparison tests. Statistical significance was indicated when *p* < 0.05. After measurement of the ear-swelling response, mice were euthanized by cervical dislocation following an ethically approved way by the Institutional Committee.

## Results

### Effect of capsaicin, piperine and PC extract on human TRPV1 activation

**Fig. 2:**
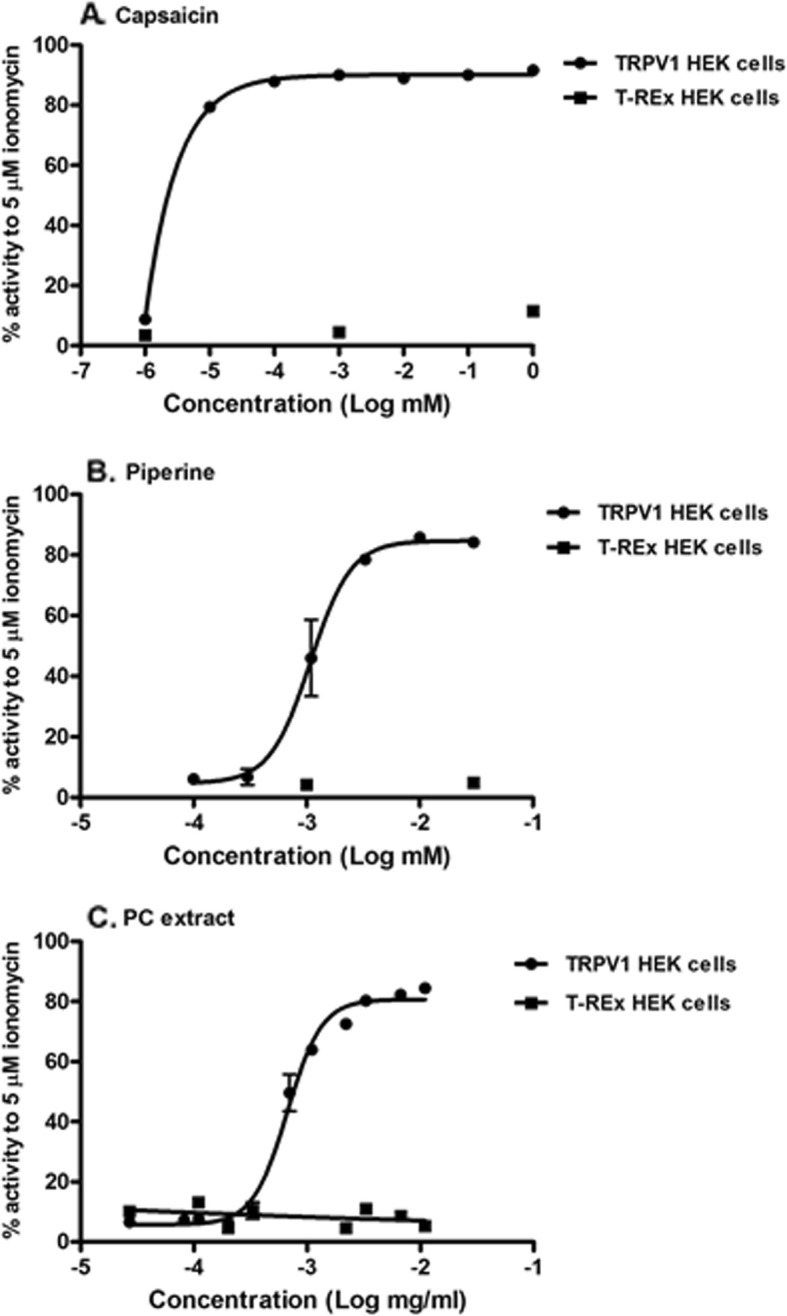
Calcium influx responses into TRPV1-expressing HEK cells or T-REx HEK cells. The cells were treated with various concentration of capsaicin (**a**), piperine (**b**) or *P. chaba* extract (**c**). The values represent percent response relative to that treated with 5 μM ionomycin. Data are mean ± SEM (*n* = 2)

**Fig. 3:**
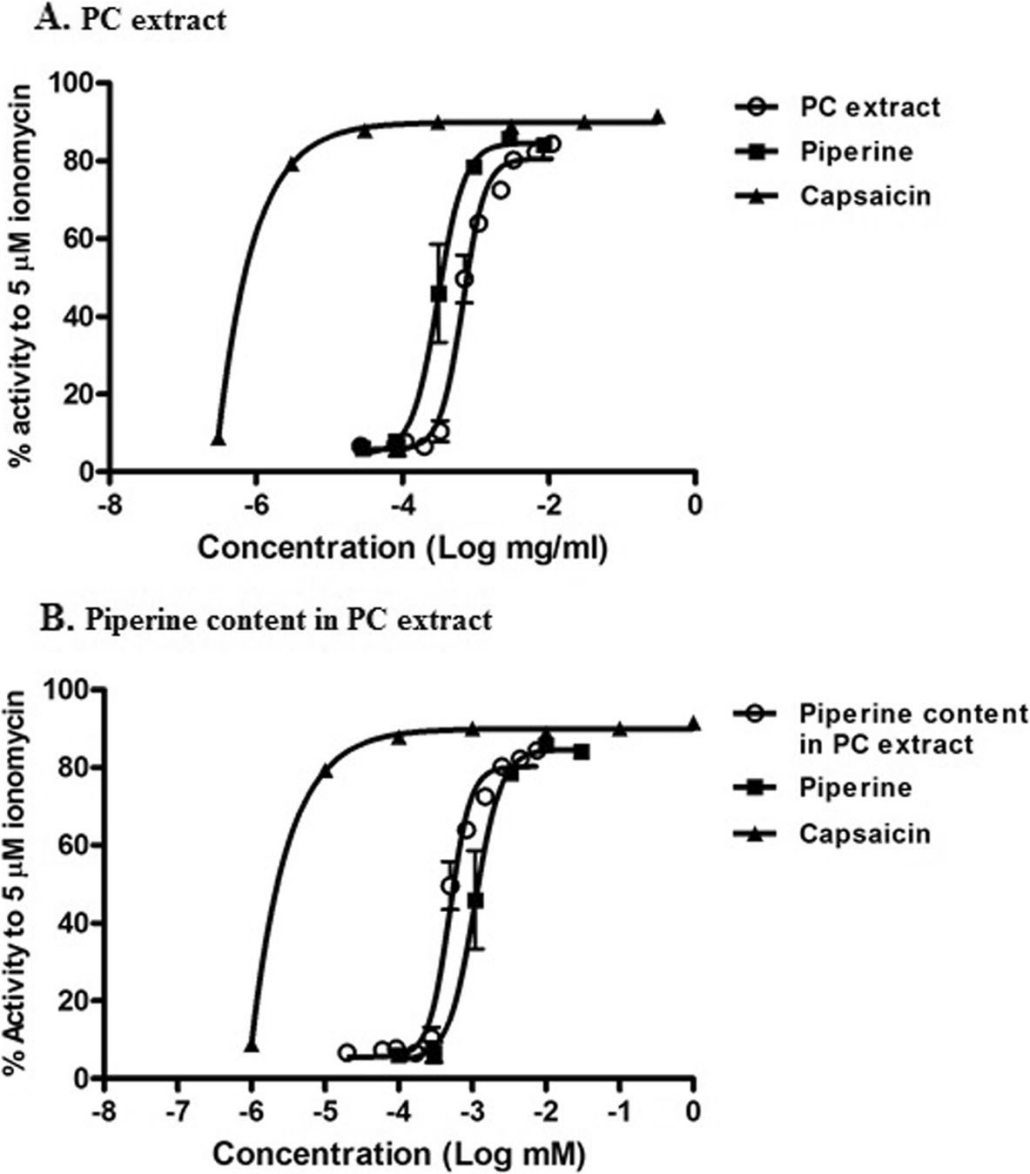
Comparison of the effects of PC extract (**a**) and piperine content in the PC (**b**) extract, on TRPV1 activation. Data are plotted on w/v basis (abscissa). Data are mean ± SEM (*n* = 2)

TRPV1-expressing HEK293 cells were treated with capsaicin, piperine or PC extract at various concentrations. The results show that capsaicin (TRPV1 agonist, positive control), piperine and PC extract increased calcium influx into TRPV1 HEK293 cells with EC_50_ value of 1.14 ng/ml (3.73 nM), 0.31 μg/ml (1.08 μM) and 0.67 μg/ml, respectively (Table 1). Moreover, all of samples had no effect on T-Rex HEK293 cells as control cells without expression of TRPV1, as shown in Fig. 2. From Table 1, we found that the EC_50_ values of piperine and capsaicin were lower than that of PC extract by 2.16 and 587.72 fold, respectively, on a weight basis. The results in Fig. 3a showed that capsaicin had the strongest activity on TRPV1 activation. Furthermore, pure piperine showed higher activity on TRPV1 channel than PC extract.

**Table 1:** The EC_50_ value of capsaicin, piperine and PC extract on TRPV1 activation

Samples	EC_**50**_ value
PC extract	0.67 μg/ml
Piperine	0.31 μg/ml(1.08 μM)
Capsaicin	1.14 ng/ml(3.73 nM)

We compared effects of pure piperine and PC extract based on the piperine content in the extract. As shown in Fig. 3b, piperine increased calcium response in TRPV1-expressing HEK293 cells with EC_50_ value of 1.08 μM. In contrast, PC extract showed even stronger increase of calcium influx in TRPV1-expressing HEK293 cells with EC_50_ value of 0.49 μM piperine based on the calculation of piperine content in the PC extract.

### TRPV1 antagonist inhibited calcium influx in TRPV1-expressing HEK cells induced by piperine and PC extract

**Fig. 4:**
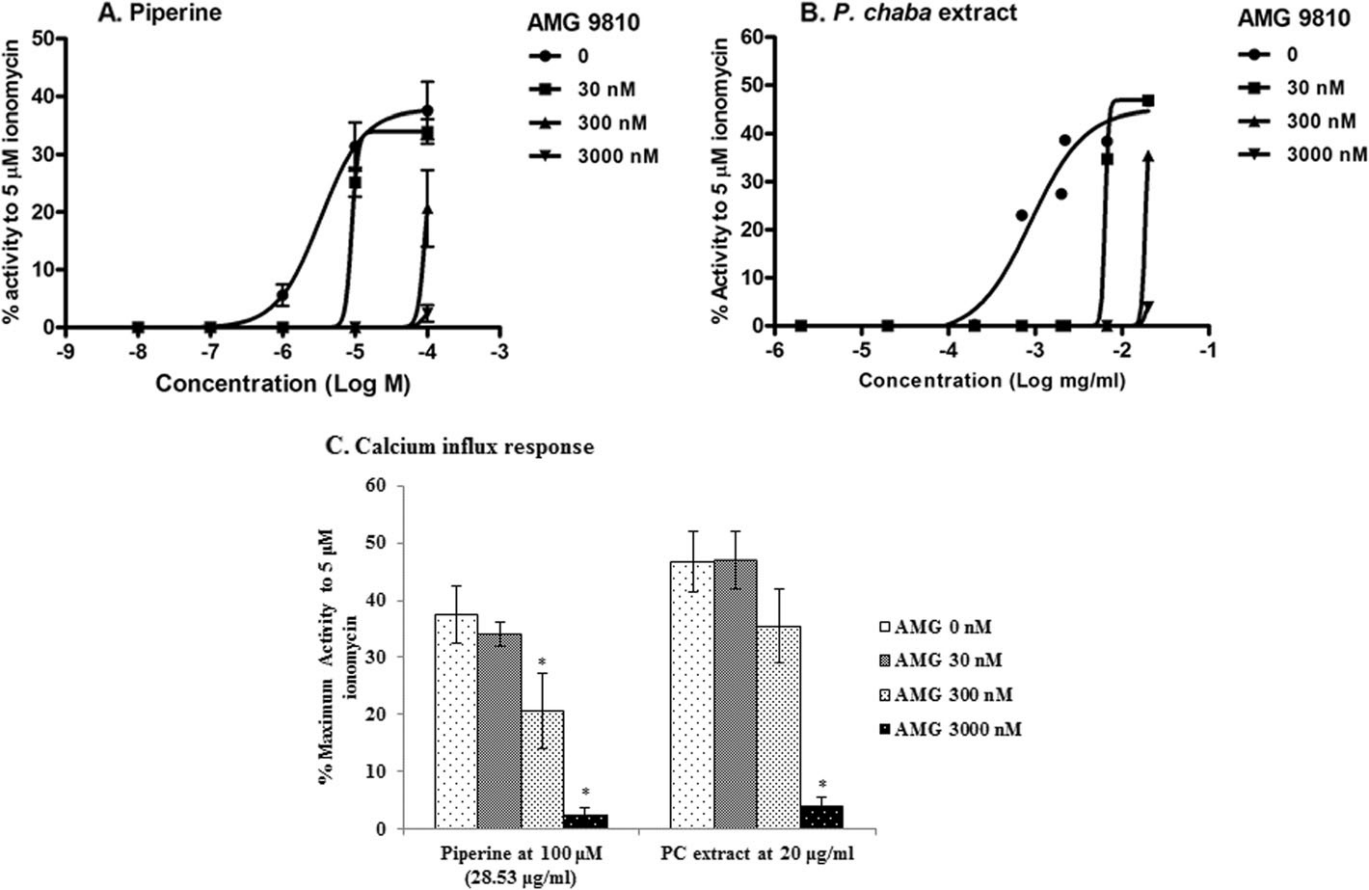
TRPV1 antagonist AMG9810 inhibited TRPV1 activation by piperine (**a**) or *P. chaba* extract (**b**). Calcium influx responses (relative to the response to 5 μM ionomycin) are shown in the presence of various concentrations of AMG 9810 or in its absence (**c**). Concentrations of TRPV1 agonists are shown in the abscissa. The response is detected in the presence of various concentrations of AMG 9810 as shown in the panels. Data are mean ± SEM (*n* = 2). * *p* < 0.05 when compared with AMG 0 nM

TRPV1-expressing HEK293 cells were treated with various concentrations of piperine or PC extract in the presence of AMG9810 (TRPV1 antagonist) at concentrations of 30, 300 and 3000 nM. The results show that the activity of piperine and PC extract, defined by the percentages of the maximal calcium responses caused by 5 μM ionomycin treatments, decreased in the presence of AMG9810 at concentrations of 30 and 300 nM. AMG9810 at the highest concentration (3000 nM) almost completely inhibited piperine and PC extract activity on TRPV1 receptor (Fig. 4).

### Effect of piperine and PC extraction sensitization to FITC by mouse ear swelling test

**Fig. 5:**
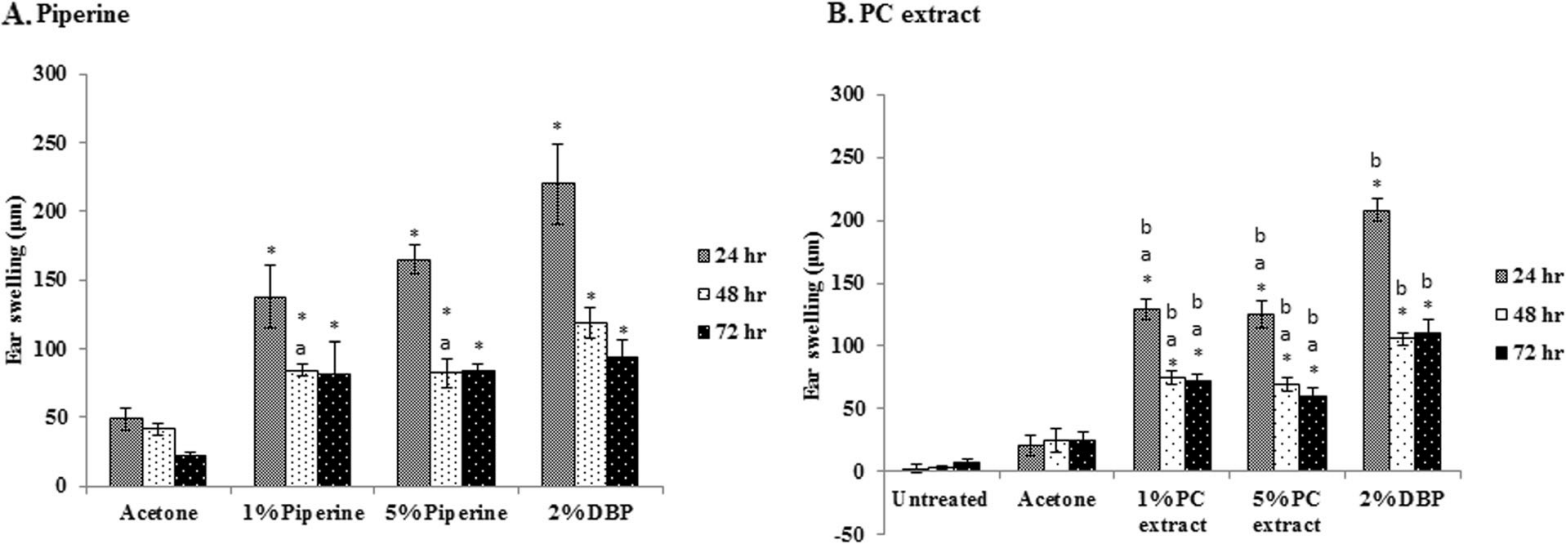
Effect of piperine (**a**) and PC extract (**b**) on the sensitization to FITC by means of mouse ear-swelling test (*n* = 6). BALB/c mice were sensitized with FITC in acetone, or FITC in 1% PCextract, 5% PC extract or 2% DBP. Mice in the untreated group were not sensitized but challenged with 0.5%FITC in 10%DBP in acetone. Mouse ear-swelling was measured at 24, 48 and 72 h after challenge.* *p* < 0.05 when compared with acetone group;^a^*p* < 0.05 when compared with 2% DBP;^b^*p* < 0.05 when compared with untreated group

The eight groups of mice did not differ in weight, age and sex, as showed in Table 2. Mouse ear swelling was measured 24, 48 and 72 h after challenge. The results show that 1% piperine and 5% piperine significantly enhanced sensitization to FITC when compared with the acetone group as revealed by ear swelling (*p*-value < 0.05). Moreover, 1% piperine and 5% piperine exhibited highest activity on mouse ear swelling at 24 h (137.50 ± 22.70, 165.00 ± 10.23 μm) and their activity decreased at 48 and 72 h with mouse ear swelling size was 81.67–84.17 μm (Fig. 5a).

**Table 2:** The body weight of mice of each group

Group	Number of mice	Body weight (g/kg)
untreated group	5	18.72 ± 0.30
Acetone group for PC	6	18.55 ± 0.43
1% PC extract in acetone	6	19.04 ± 0.30
5% PC extract in acetone	6	18.60 ± 0.39
2% DBP in acetone for PC	6	18.87 ± 0.49
Acetone group for piperine	5	17.87 ± 0.37
1% piperine in acetone	5	16.83 ± 0.45
5% piperine in acetone	5	18.15 ± 0.59
2% DBP in acetone for piperine	6	16.69 ± 0.38

PC extract (1 and 5% w/v) significantly enhanced sensitization to FITC when compared with acetone control. PC extract showed the strongest activity on mouse ear swelling at 24 h after challenge. There is no difference in the ear-swelling between groups treated with 1% PC extract or with 5% PC extract. The mouse ear swelling decreased at 48 and 72 h after challenge. The ear-swelling responses of PC extract groups were significantly lower than that of the 2% DBP group (positive control). For the untreated group, non-sensitized mice were challenged with 0.5% FITC in 10% DBP-acetone on the right auricle while 10% DBP in acetone on the left auricle. The untreated group did not exhibit FITC-dependent changes in ear thickness at 24, 48 or 72 h (Fig. 5b).

### Piperine content in the ethanolic extract of *P. chaba* Hunt.

The piperine content in PC extract, which was calculated by standard curve of piperine (R^2^ = 0.998),was 194.10 mg/g of PC extract. The chemical structure of piperine is shown in Fig. 1.

## Discussion

The transient receptor potential vanilloid 1 (TRPV1) channel consists of six transmembrane domains and a short, pore-forming hydrophobic stretch between the fifth and sixth transmembrane domains. TRPV1 channel is activated by capsaicin, noxious heat, low pH and voltage [11]. Moreover, TRPV1 receptor can be activated by compounds from plants such as capsaicin from red pepper, gingerol from ginger and piperine from pepper [13]. The role of TRPV1 channel involved in the pain transmission made TRPV1 a candidate target for analgesic effect [15]. The activation of TRPV1 lead to enhanced calcium influx. The calcium ion accumulation in cells response to desensitization of cell membrane to protect the cells from Ca^2+^ overload. The Ca^2+^ desensitization of TRPV1 channel decreased responsiveness to temperature and stimuli agents that increase analgesic effects [22]. Many natural compounds are developed as analgesic drugs (known as counterirritant drugs): for example capsaicin from red pepper and methyl salicylate from wintergreen [16, 23], whose actions are mediated by TRPV1 desensitization [24].

In this study, we focused on comparative effect of piperine and PC extract on TRPV1 receptor. PC extract contains pungent alkaloids such as piperine [3]. Piperine’s taste is sharp, peppery and leaves a burning sensation because it is reported to activate human TRPV1 receptor which is similar to the capsaicin effect [9]. We found that piperine and PC extract activated TRPV1 receptor with EC_50_ values of 0.31 μg/ml (1.08 μM) and 0.67 μg/ml, respectively when using calcium imaging assay. Piperine showed higher activated TRPV1 receptor than PC extract as 2.16 times. However, piperine content in PC extract was 19.14% w/w or piperine as 1:5 by weight of extract, it showed that some compounds or derivative of piperine as ingredients in PC extract should be synergistic effect. However, the effects of piperine and PC extract on TRPV1 receptor were less than that of capsaicin, a positive control, on TRPV1 activation. Piperine has been reported to be contained in black pepper and activated TRPV1 and TRPA1 with EC_50_ value of 0.6 μM or 0.17 μg/ml in the same method [21], which had stronger activity than this results as 1.82 times. However, piperine has also been studied on the activation of TRPV1 receptor (EC_50_ = 33.3 μM) and simultaneously on the modulation of GABA_A_ receptors by using the different method as the two-microelectrode-voltage-clamp technique [10]. Thus, the effects of PC extract and piperine are specific for TRPV1 receptor because their effects are inhibited in the presence of a TRPV1 antagonist, AMG9810, as shown in Fig. 5.

The effects of pure piperine compound in PC extract in terms of piperine content in the extract are shown in Fig. 4. The results show that the PC extract samples with lower piperine content gave rise to a stronger response than the pure piperine sample, which may mean that TRPV1 receptor was activated not only by piperine but also by some other compounds in the PC extract. Previous work showed that other chemical compounds of *P. chaba* Hunt. (such as brachystamide B, dehydropipernonaline, fragaramide, guineensine, methyl piperate, isopiperine, isochavicine, piperonal, piperlonguminine and retrofractamide) could activate TRPV1 channel [21]. These compounds in PC extract may show synergistic effect on TRPV1 activation.

In animals, we investigated the effect of the adjuvant effect of 1% piperine, 5% piperine, 1% PC extract and 5% PC extract on contact sensitization by using an FITC-induced contact hypersensitivity model using ear swelling response [18]. Our results have shown that piperine increased ear swelling in this animal model. Moreover, adjuvant effect of 5% piperine was fractionally higher than the adjuvant effect of 1% piperine. For PC extract, the 5% PC extract contains approximately 1% piperine, thus the 1% PC extract contains 0.2% of piperine. Yet, the adjuvant effect of 1% PC extract was similar to the adjuvant effect of 5% PC extract. These results suggest that the adjuvant effect of PC extract reached saturation at 1%.

Phthalate esters, such as DBP, which exhibited an enhancing effect on FITC-induced contact hypersensitivity, were shown to activate TRPV1 as well as TRPA1 cation channels [19]. Although several TRPA1 agonists have been shown to enhance FITC-induced contact hypersensitivity [20], TRPV1 agonists have not been examined. Thus, this is the first report showing that a TRPV1 agonist (piperine) and a natural product (PC extract) can activate TRPV1 to exhibit an immunomodulatory activity by means of an FITC-induced contact hypersensitivity mouse model.

## Conclusions

The present study indicates that PC extract and its major compound (piperine) activated TRPV1 channel. Piperine is not the only active compound in PC extract which activated TRPV1 channel but other compounds in PC extract also show adjuvant effect and activation of TRPV1 channel. Future studies should be focused on the effect of PC extract on TRPV1 desensitization for confirmation of its analgesic effect. However, the development of PC extract should be performed carefully to produce an analgesic drug, because of this extract induced contact hypersensitivity in mice, such as drugs used as counterirritant which piperine was used as main ingredient for analysis.

## Data Availability

The datasets used and/or analysed during the current study available from the corresponding author on reasonable request. Datasets consist of 4 parts: Part 1: Calcium influx responses into TRPV1-expressing HEK cells or T-REx HEK cells. Part 2: TRPV1 antagonist AMG9810 inhibited TRPV1 activation by piperine or *P. chaba* extract. Part 3: Effect of piperine on the sensitization to FITC by means of mouse ear-swelling test. and Part 4: Effect of PC extract on the sensitization to FITC by means of mouse ear-swelling test.

## Abbreviations

TRPV1: Transient receptor potential cation channel subfamily V member 1
PC: *Piper chaba*
FITC: Fluorescein isothiocyanate
CHS: Contact hypersensitivity
DBP: Dibutylphthalate
